# Weight and Lifestyle Behavior Changes in Chinese Health Care Workers During the COVID-19 Pandemic: 3-Year Retrospective Survey

**DOI:** 10.2196/50754

**Published:** 2024-12-10

**Authors:** Xinyue Guo, Shaoqing Gong, Ying Chen, Xiaohui Hou, Tong Sun, Jianqiang Wen, Zhiyao Wang, Jingyang He, Xuezhu Sun, Sufang Wang, Zhixin Chen, Xue Feng, Xiangyang Tian

**Affiliations:** 1 Beijing Center for Disease Prevention and Control Beijing China; 2 Luohe Medical College Luohe China; 3 Department of Nutrition and Food Hygiene School of Public Health Anhui Medical University Anhui, Hefei China; 4 Chinese Center for Health Education Beijing China; 5 Shandong Center for Disease Control and Prevention Shandong China; 6 Gansu Province Traditional Chinese Medicine Development Center Gansu China; 7 Health Promotion and Education Center of Xinjiang Uygur Autonomous Region Xinjiang Uygur Autonomous Region China; 8 Health Education Institute of Henan Center for Disease Control Henan China; 9 Health Lifestyle Medical Center Fuwai Hospital Chinese Academy of Medical Sciences Beijing China

**Keywords:** COVID-19, healthcare workers, lifestyle behavior, overweight, obesity, physical activity, mental health, stress, anxiety,depression, pandemic

## Abstract

**Background:**

Health care workers (HCWs) played a key role in preventing and controlling COVID-19. Higher infection risks and intensive work led to occupational burnout for many HCWs, which may affect their lifestyle behaviors and weight.

**Objective:**

This study aimed to assess HCWs’ self-rated health status, overweight and obesity rates, lifestyle behaviors, and psychoemotional changes from 2019 to 2022 across China and to analyze the factors associated with changes from underweight or normal weight in 2019 to overweight or obese in 2022.

**Methods:**

In this retrospective study, 100 health care institutions were randomly selected from 5 provinces or regions in China. All HCWs who worked in the institutions for at ≥3 years were invited to complete the electronic questionnaire and participate in the online survey from August 1, 2022, to August 31, 2022. Collected data included changes in lifestyle behaviors (dietary habits, physical activity, sleep quality, smoking, alcohol consumption), psychoemotional conditions (persistent stress or recurrent anxiety or depressed mood), health status, and chronic disease control from December 2019 to August 2022. Height and weight in 2019 and 2022 were retrieved from annual physical examination records. Overweight and obesity were defined as 24.0 kg/m^2^≤BMI<28.0 kg/m^2^ (overweight) and BMI≥28.0 kg/m^2^ (obesity). Chi square tests and ANOVAs were used to assess the associations between groups. Logistic regression models were used to analyze the factors associated with HCWs becoming overweight or obese from 2019 to 2022.

**Results:**

The questionnaire was submitted by 23,234 HCWs. Of the underweight or normal weight HCWs in 2019, 12.67% (1486/23,234) became overweight or obese in 2022; this change was associated with the following factors: 34-43 years old (OR 0.843, 95% CI 0.740-0.960), 44-53 years old (OR 0.738, 95% CI 0.635-0.960), and 54-63 years old (OR 0.503, 95% CI 0.368-0.685; reference: 24-33 years old), reduction in or never or rarely engaging in physical activity (OR 1.201, 95% CI 1.055-1.368; reference: increase in physical activity; *P*=.006), increased appetite (OR 2.043, 95% CI 1.788-2.034; reference: reduction or no change in appetite; *P*<.001). From 2019 to 2022, 51.29% (11,917/23,234) of the respondents experienced increased persistent stress or recurrent anxiety or depressed mood; 44.38% (10,311/23,234) stayed up late more often. Increased persistent stress or recurrent anxiety or depressed mood was associated with physical activity (OR 0.421, 95% CI 0.398-0.447; *P*<.001) and appetite (OR 1.601, 95% CI 1.483-1.728; *P*<.001).

**Conclusions:**

The pandemic was associated with overweight and obesity for HCWs due to changes in lifestyle behaviors, especially reduced physical activity and increased appetite related to increased persistent stress or recurrent anxiety or depressed mood caused by excessive workload. An integrated approach is needed to address overweight and obesity and lifestyle changes among HCWs by releasing negative psychoemotional conditions through workload reduction in future stressful events.

## Introduction

Health care workers (HCWs) have played a key role in the COVID-19 pandemic control since the end of 2019. The high risk of infection [1], long-term isolation, and intensive work not only caused burnout for many HCWs but also substantially affected their mental health [2], daily routine [3,4], physical activity, diet, and other lifestyle behaviors [5] and has put them at high risk for obesity [6-9]. A literature review in 2020 showed that HCWs gained weight and changed their lifestyles to varying degrees during the pandemic [10]. A study in the United States on lifestyle behaviors and weight changes among frontline HCWs since the COVID-19 outbreak [11] showed that dietary patterns of HCWs during the pandemic (January 2021) were negatively impacted, with an increase in snack, fast food, take-away food, and alcohol consumption and an average weight increase of 2.99 pounds compared with prepandemic levels (March 2021). Jin et al [12] found that 39.7% were less physically active and 36% had less sleep in a survey of 589 Chinese HCWs. A study by Zhang et al [13] surveyed the Chinese frontline medical staff who supported Hubei Province during the initial stage of the pandemic and found that most had an unbalanced diet and 26.2% gained weight. A survey of Brazilian doctors by Gomes et al [6] found that 60% had less physical activity, 39.9% consumed more alcohol, and 32.9% gained weight.

Being overweight or obese and lifestyle behavior changes increase the risk of various chronic diseases, such as hypertension, type 2 diabetes mellitus, coronary heart disease, stroke, certain cancers, and osteoporosis [14-20]. Being overweight or obese can directly affect daily activities or even lead to mental and emotional disorders [21], impairing work performance [22], increasing the absenteeism rate due to illness and frequent overtime [23-25], and increasing occupational injuries, and can endanger the sustainable development of health care institutions [26].

Basically, overweight and obesity result from consuming more calories than expended [27]; hence, any factor that can increase food calorie intake and reduce exercise expenditure may lead to overweight and obesity. Studies indicated that occupational factors such as working hours, work intensity, work stress, and shifts are associated with overweight or obesity in HCWs [28-31]. Shift nurses are more likely to consume excessive food, have more coffee and snacks, and participate in less exercise during night shifts [32-34], which further increases the likelihood of overweight or obesity. Negative psychoemotional conditions (eg, chronic stress, anxiety, and depression) can have a substantial impact on a person’s diet and physical activity [35-37]. According to a survey by the American Psychological Association, 39% of adults cope with stress by overeating and eating unhealthy foods [35]. A longitudinal study of nearly 1400 women in Australia showed an association between higher stress and less physical activity and more frequent consumption of fast food [36]. This is due to the fact that constant stress can activate the hypothalamic-pituitary-adrenal axis in humans, promote cortisol secretion, affect sleep, and increase the intake of “self-rewarding foods” (high in fat and sugar), thereby increasing the incidence of obesity, especially in the abdomen [37].

HCWs are considered to experience a heavier workload and mental stress than their counterparts in many other industries due to their longer working hours, less personal time, and higher burnout rates, possibly making them more susceptible to the risk of overweight or obesity [38]. Over the past 3 years, the extra burden from the COVID-19 pandemic has been substantial for HCWs. A systematic review and meta-analysis of anxiety-related symptoms among HCWs affected by the pandemic reported that 42% showed characteristics of anxiety, 40% experienced acute stress, 37% experienced burnout, and 32% had posttraumatic stress disorder [39].

There have been many studies on the impact of the pandemic on weight changes and lifestyle behaviors in the general population [11,40-42]; however, few focused exclusively on HCWs nationwide in China. Weight, lifestyle behavior, and psychoemotional changes and the relationship between them among the Chinese HCWs remain unknown.

The aim of this study was to assess HCWs’ self-rated health status, overweight and obesity rates, lifestyle behaviors, and psychoemotional changes from 2019 to 2022 across China as well as to analyze the factors associated with weight changes resulting in them moving from underweight or normal weight in 2019 to overweight or obese in 2022.

## Methods

### Study Design

This study was based on a retrospective anonymous web survey [43] using a structured self-administered questionnaire and was conducted in accordance with the Checklist for Reporting Results of Internet E-Surveys (CHERRIES) [44]. The respondents were randomly sampled, and relevant data were collected via recall, including sociodemographic characteristics, health status, and height and weight in 2019 and 2022, as well as changes in lifestyle behaviors and psychoemotional states from 2019 to 2022.

### Study Population, Sampling Strategy, and Eligibility Criteria

A multistaged, clustered, random sampling method was used. First, 5 provincial administrative regions (Beijing City, Shandong Province, Henan Province, Gansu Province, and Xinjiang Uygur Autonomous Region) were selected at random from 32 provinces, autonomous regions, and municipalities of China. Second, 20 health care institutions were sampled from each of the 5 provincial administrative regions considering interprovincial differences and intraprovincial homogeneity of the institutions. Third, HCWs from the selected institutions who met the inclusion and exclusion criteria were included. A total of 100 medical institutions were sampled. The survey was conducted from August 1, 2022, to August 31, 2022.

The inclusion criteria were as follows: (1) age ≥24 years, (2) working in the health care institutions for at least 3 years from 2019 to 2022, (3) possessing and using a smartphone with the WeChat app, and (4) providing informed consent and voluntarily participating in the survey.

### Sample Size Estimation

The sample size of this study was estimated by using the formula for staged clustered random sampling:



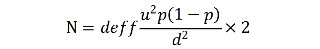



where *p*=6.5% (estimated obesity rate among HCWs) [45], *d* is the absolute error and was calculated as 10% (error tolerance)×6.5%, *u*=1.96, *deff* is the design efficiency with a value of 2.0, and sampling was stratified into urban and rural areas (×2). Considering nonresponse, an additional 10% of the calculated sample size was added, leading to a required sample size of 24,314 HCWs.

### Survey Tools

The survey questionnaire was developed purposely by the study group to collect data. First, we pooled the items based on a literature review and expert interviews. Second, a nominal group discussion with 10 senior experts in lifestyle was organized, and only the items (questions) with face validity above 0.8 were included [46,47]. Third, we invited 10 HCWs to conduct a pretest using the initial questionnaire to validate all the items. The weak items were removed or revised. The final questionnaire was comprised of the following modules: informed consent, answering guide, sociodemographic information, general health status (self-rated, presently and in 2019), height (m) and weight (kg) in 2019 and 2022 (based on their annual physical examination record), and changes in lifestyle behaviors (dietary habits, physical activity, sleep quality, smoking, alcohol consumption, and persistent stress or recurrent anxiety or depressed mood) from 2019 to 2022.

Sociodemographic information included gender, age, education, and working and living locations (urban or rural). General health status was self-rated, and presence of chronic diseases (eg, diagnosed hypertension, cardiovascular and cerebrovascular diseases, cancer, diabetes, chronic obstructive pulmonary disease, osteoporosis [48,49]) was compared with that in 2019. Dietary habits included consumption of take-out food (generally regarded as ultraprocessed), vegetables and fruits, fried food, snacks or desserts, and sugar-sweetened beverages as well as breakfast frequency. Data about sleep quality (staying up late [not falling asleep after 12 AM due to specific affairs, voluntarily or involuntarily [50]] and sleep duration) were also collected. Overweight or obese was based on BMI (BMI=weight [kg]/height [m]^2^); underweight was defined as BMI<18.5 kg/m^2^, normal weight was defined as 18.5 kg/m^2^≤BMI<24.0 kg/m^2^, overweight was defined as 24.0 kg/m^2^≤BMI<28.0 kg/m^2^, and obese was defined as BMI≥28.0 kg/m^2^ [51].

To measure psychoemotional states, we defined persistent stress with the following question: “Have frequently experienced feelings of being under too much mental or emotional pressure made one angry/irritated/moody/frustrated, worried, or unable to sleep for at least 6 months?” We defined recurrent anxiety with the following statement: “has experienced recurrent feelings of being nervous or restless or a sense of impending danger, panic, or doom with increased heart rate/sweating/ accelerated breathing/trembling for at least 6 months.” We defined recurrent anxiety or depressed mood with the following statement: “has experienced persistent feelings of sadness, tearfulness, emptiness or hopelessness, and loss of interest or pleasure in most or all normal activities, such as sex, hobbies or sports, with angry outbursts, irritability or frustration, even over small matters for at least 6 months.”

Each item (question) was rated on a 5-point Likert scale [52] using the responses “great increase/great improvement,” “increase/improvement,” “no change,” “reduction/deterioration,” “great reduction/great deterioration,” or “never or rarely” or “no diagnosed such disease.” Except for height and weight, which are continuous variables, all other variables in this study are categorical. In order to increase the statistical power, we merged 3 psychoemotional states into 1 variable of “persistent stress and/or recurrent anxiety/depressed mood” for analysis, and an increase in any of the 3 conditions were considered to increase persistent stress or recurrent anxiety or depressed mood.

The Cronbach α of the final questionnaire was 0.82, with 3 factors explaining 63.55% of the total variance based on exploratory factorial analysis [53].

### Data Collection

The Wenjuanxing Web Survey System (an online questionnaire research platform in Chinese) [54] was used to generate a QR code for the electronic questionnaire. The investigators contacted the liaison of the selected health care institutions and sent the QR code to all the HCWs at the institution. The respondents were required to scan or press the QR code through WeChat to fill out the electronic questionnaire and submit it to the Wenjuanxing backstage after completion. Their WeChat ID was used to ensure that each respondent could only submit the questionnaire once.

Participants’ responses were anonymous and confidential. The anonymity and confidentiality policy was declared at the beginning of the electronic questionnaire. The respondents were also informed that data would be used for research purposes only. Participants completed the questionnaire when directly connected to the Wenjuanxing platform. In total, 25,000 questionnaires were sent out, and 24,344 questionnaires were returned, with a recovery rate of 97.38%. Each submitted questionnaire was reviewed, and questionnaires with incomplete or missing items, incorrect information, and obvious logical errors were regarded as invalid (1110/24,344, 4.56%); 23,234 questionnaires entered the final analysis.

### Statistical Analysis

Statistical analysis was performed using SPSS 25.0 (IBM Corp) software. The sociodemographic characteristics were analyzed using descriptive statistics, and the quantitative data are represented by mean (SD). Chi square tests were used to assess the association between categorical variables and trends within a categorical variable. ANOVAs were performed to compare continuous variables among 2 or more groups. Multivariate binary logistic regression was used to test the independent variables (changes in sociodemographic characteristics, lifestyle behaviors, and psychoemotional states) related to the BMI changes, and a multivariate binary logistic regression model was constructed taking the identified statistically significant lifestyle behaviors as dependent variables and “persistent stress and/or recurrent anxiety/depressed mood” as the independent variable. *P*<.05 was considered statistically significant for all analyses.

### Ethical Considerations

This study was conducted in compliance with the Declaration of Helsinki and was reviewed and approved by the ethics committee of Fuwai Hospital, Chinese Academy of Medical Sciences (number 2021-1559). Informed consent was obtained from participants in accordance with ethical guidelines. This study abided by the principles of scientific research ethics, clearly stated the purpose of this survey in the preface of the electronic questionnaire, and strictly protected the privacy of all respondents. All respondents were required to be honest in their responses. Participants were able to stop answering and leave the questionnaire at any stage before the end of the process, with no answers being saved. Respondents’ answers were saved just by clicking on the “submit” button. Upon completing the survey, participants acknowledged their voluntary consent to participate in this anonymous study. Every participant could obtain daily necessities as rewards upon submission, such as napkins, towels, soap, and hand sanitizer.

## Results

### Sociodemographic Characteristics of the Respondents

Of the 23,234 HCWs, 31.8% (7388/23,234) were male, and 68.2% (15,846/23,234) were female. The average age was 39.85 (SD 9.41; range 24-80) years, and more than 60% (14,804/23,234, 63.72%) were in the following age groups: 34-43 years and 44-53 years. In total, 73.78% (17,143/23,234) of the respondents had an education at the college or university level, and 61.44% (14,276/23,234) worked and lived in urban areas. See Table 1.

**Table 1:** Sociodemographic characteristics of the respondents (N=23,234).

Sociodemographic characteristics		Results, n (%)
**Gender**		
	Male	7388 (31.8)
	Female	15,846 (68.2)
**Age (years)^a^**		
	24-33	6768 (29.13)
	34-43	7895 (33.98)
	44-53	6909 (29.74)
	54-63	1405 (6.05)
	≥64	222 (0.96)
**Education level**		
	Junior high school or less	247 (1.06)
	Senior high school or secondary specialized school	5138 (22.11)
	College or undergraduate degree	17,143 (73.78)
	Master’s degree or higher	706 (3.04)
**Working and living location**		
	Urban	14,276 (61.44)
	Rural	8958 (38.56)

^a^35 missing responses.

### Health Status of the Respondents

From 2019 to 2022, 31.19% (7247/23,234) of the respondents reported that their health condition deteriorated; meanwhile, 18.76% (4359/23,234) reported that it improved, and 50.05% (11,628/23,234) reported no change. In total, 22.45% (5215/23,234) had chronic diseases, of which 66.17% (3451/5215) reported their disease was better controlled and 33.83% (1764/5215) reported their disease was less controlled.

### BMI Changes From 2019 to 2022

In 2019, the mean BMI of the participants was 23.63 (SD 3.48) kg/m^2^; 32.26% (6651/20,619) were overweight, and 10.84% (2236/20,619) were obese. In 2022, the mean BMI was 23.85 (SD 3.42) kg/m^2^; 34.26% (7403/21,608) were overweight, and 11.22% (2424/21,608) were obese. The difference in mean BMI between 2019 and 2022 was statistically significant (*P*<.001). Nevertheless, the proportion of those who were overweight or obese increased by 2% (*P*<.001) and 0.38% (*P*<.001), respectively (Table 2).

**Table 2:** Sociodemographic characteristics by BMI categories in 2019 and 2022.

Sociodemographic characteristics		BMI (kg/m^2^), mean (SD)		*P* value^a^	Underweight, n (%)		Normal weight, n (%)		Overweight, n (%)		Obese, n (%)		*P* value^b^
		2019^c^	2022^d^		2019^c^	2022^d^	2019^c^	2022^d^	2019^c^	2022^d^	2019^c^	2022^d^	
**Gender**				<.001									<.001
	Male	25.39 (3.43)	25.50 (3.33)		101 (1.5)	88 (1.26)	2188 (32.49)	2159 (31)	3073 (45.63)	3317 (47.63)	1372 (20.37)	1400 (20.1)	
	Female	22.78 (3.17)	23.06 (3.18)		938 (6.76)	826 (5.64)	8505 (61.25)	8708 (59.46)	3578 (25.77)	4086 (27.9)	864 (6.22)	1024 (6.99)	
**Age (years)^e^**				<.001									<.001
	24-33	22.08 (3.50)	22.63 (3.60)		674 (12.09)	564 (9.35)	3569 (63.99)	3690 (61.16)	981 (17.59)	1315 (21.8)	353 (6.33)	464 (7.69)	
	34-43	23.71 (3.39)	23.88 (3.36)		264 (3.66)	234 (3.13)	3842 (53.31)	3844 (51.47)	2313 (32.09)	2536 (33.96)	788 (10.93)	854 (11.44)	
	44-53	24.61 (3.15)	24.69 (3.10)		80 (1.26)	96 (1.46)	2731 (43.01)	2727 (41.56)	2664 (41.95)	2859 (43.57)	875 (13.78)	880 (13.41)	
	54-63	25.00 (3.04)	24.92 (2.95)		13 (1.02)	13 (0.98)	479 (37.57)	523 (39.5)	582 (45.65)	586 (44.26)	201 (15.76)	202 (15.26)	
	≥64	24.77 (2.81)	24.60 (2.79)		5 (2.54)	5 (2.45)	66 (33.5)	73 (35.78)	107 (54.31)	102 (50)	19 (9.64)	24 (11.76)	
**Education level**				<.001									<.001
	Junior high school or less	24.71 (3.31)	24.80 (3.19)		7 (3.32)	5 (2.25)	79 (37.44)	83 (37.39)	94 (44.55)	105 (47.3)	31 (14.69)	29 (13.06)	
	Senior high school or secondary specialized school	24.66 (3.30)	24.73 (3.24)		89 (1.91)	100 (2.07)	1917 (41.21)	1947 (40.38)	1931 (41.51)	2047 (42.45)	715 (15.37)	728 (15.1)	
	College or undergraduate degree	23.32 (3.48)	23.58 (3.45)		917 (6.07)	787 (4.95)	8311 (55.04)	8454 (53.21)	4430 (29.34)	5038 (31.71)	1443 (9.56)	1609 (10.13)	
	Master’s degree or higher	23.21 (3.16)	23.53 (3.10)		26 (3.97)	22 (3.25)	386 (58.93)	383 (56.66)	196 (29.92)	213 (31.51)	47 (7.18)	58 (8.58)	
**Working and living location**				<.001									<.001
	Urban	23.16 (3.37)	23.43 (3.34)		791 (6.28)	679 (5.12)	7137 (56.64)	7296 (55.05)	3646 (28.93)	4113 (31.03)	1027 (8.15)	1165 (8.79)	
	Rural	24.38 (3.52)	24.51 (3.44)		248 (3.09)	235 (2.81)	3556 (44.35)	3571 (42.74)	3005 (37.48)	3290 (39.38)	1209 (15.08)	1259 (15.07)	
Total sample		23.63 (3.48)	23.85 (3.42)		1039 (5.04)	914 (4.23)	10,693 (51.86)	10,867 (50.29)	6651 (32.26)	7403 (34.26)	2236 (10.84)	2424 (11.22)	<.001

^a^ANOVA of the rates of overweight or obesity between 2019 and 2022.

^b^Chi square analysis of the rates between 2019 and 2022.

^c^2615 missing.

^f^1626 missing.

^e^35 missing.

The mean BMIs of males in 2019 and 2022 were 25.39 (SD 3.43) kg/m^2^ and 25.50 (SD 3.33) kg/m^2^, respectively, and both were higher than those of females (mean 22.78, SD 3.17 kg/m^2^ in 2019 and mean 23.06, SD 3.18 kg/m^2^ in 2022). The male overweight and obesity rates in 2019 and 2022 were 66.01% (4445/6734) and 67.73% (4717/6964), respectively, and both were higher than those of women (4442/13,885, 31.99% in 2019 and 5110/14,644, 34.89% in 2022). The proportions of overweight or obesity in those aged ≥64 years (126/197, 63.95% in 2019 and 126/204, 61.76% in 2022) and with a junior high school education or less (125/211, 59.24% in 2019 and 134/222, 60.36% in 2022) were higher than those of the other age groups and education groups, respectively. The overweight and obesity rates for those working and living in rural areas (4214/8108, 52.56% in 2019 and 4549/8355, 54.45% in 2022) were higher than those working and living in urban areas (Table 2).

From 2019 to 2022, 12.67% (1486/11,732) of underweight or normal weight respondents became overweight or obese, and the rates were higher for men (425/2289, 18.57%) and those in rural areas (536/3804, 14.09%) than for women (1061/9443, 11.24%) and those in urban areas (950/7928, 11.98%), respectively. In total, 10.51% (934/8887) of overweight or obese respondents became underweight or normal weight; the rates were higher for women (638/4442, 14.36%) and those in urban areas (570/4673, 12.2%) than for men (296/4445, 6.65%) and those in rural areas (364/4214, 8.64%), respectively. Additionally, a statistically significantly higher proportion of overweight or obese respondents aged 24-33 years (213/1334, 15.97%) became underweight or normal weight than the other age groups. With an increase in education level, the proportion who moved from overweight or obese to underweight or normal weight increased. All of these differences were statistically significant (Table 3).

**Table 3:** Proportion of participants who changed BMI from 2019 to 2022 (N=23,234).

Sociodemographic characteristics		Underweight or normal weight to overweight or obese, n (%)	*P* value	Overweight or obese to underweight or normal weight, n (%)	*P* value
**Gender**			<.001		<.001
	Male	425 (18.57)		296 (6.65)	
	Female	1061 (11.24)		638 (14.36)	
**Age (years)^a^**			.26		<.001
	24-33	520 (12.26)		213 (15.97)	
	34-43	523 (12.74)		348 (11.22)	
	44-53	383 (13.63)		291 (8.22)	
	54-63	51 (10.37)		70 (8.94)	
	≥64	9 (12.68)		12 (9.52)	
**Education level**			.66		<.001
	Junior high school or less	9 (10.47)		7 (5.6)	
	Senior high school or secondary specialized school	269 (14.76)		218 (8.24)	
	College or undergraduate degree	1154 (12.51)		677 (11.53)	
	Master’s degree or higher	54 (13.11)		32 (13.17)	
**Working and living location**			<.001		<.001
	Urban	950 (11.98)		570 (12.2)	
	Rural	536 (14.09)		364 (8.64)	
Total sample		1486 (12.67)		934 (10.51)	

^a^35 missing responses.

### Lifestyle Behavior and Psychoemotional Changes for Chinese HCWs From 2019 to 2022

**Figure 1:**
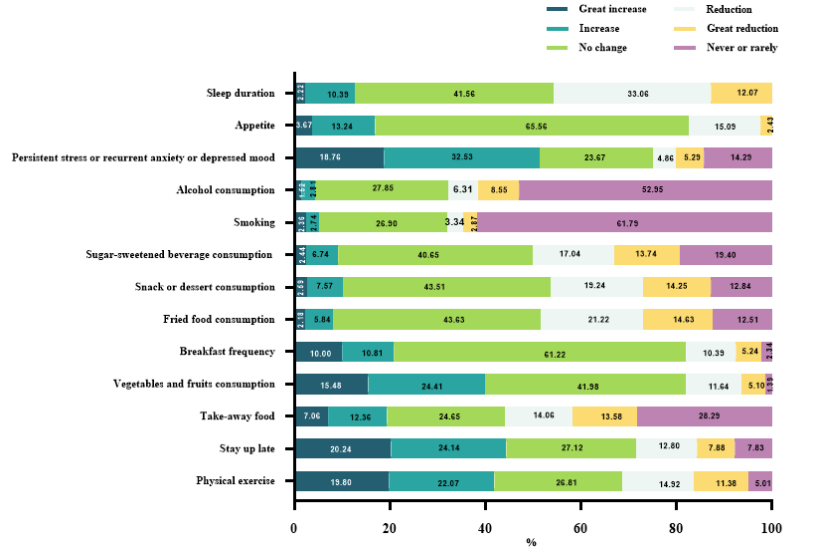
Lifestyle and psychoemotional changes of respondents from 2019 to 2022.

The proportions of respondents with a great increase or increase in physical activity, vegetable and fruit consumption, breakfast frequency, staying up late, and persistent stress or recurrent anxiety or depressed mood were statistically significantly higher than those of respondents with a reduction or great reduction in these factors. Of the respondents, 51.29% (11,917/23,234) reported a great increase or increase in persistent stress or recurrent anxiety or depressed mood. The top 3 lifestyle behaviors with a “great increase/increase” were staying up late (10,311/23,234, 44.38%), physical activity (9729/23,234, 41.87%), and vegetable and fruit consumption (9268/23,234, 39.89%). The proportions of respondents with a great increase or increase in their consumption of take-out food, fried food, snacks or desserts, and sugar-sweetened beverages; cigarette smoking; alcohol consumption; and sleep duration were significantly lower than those with a reduction or great reduction. The top 3 lifestyle behaviors that were reduced or greatly reduced were sleep duration (10,648/23,234, 45.83%), fried food consumption (8328/23,234, 35.84%), and sugar-sweetened beverage consumption (7781/23,234, 33.49%). All of these differences were statistically significant (Figure 1).

### Univariate Analyses of Lifestyle Behavior and Psychoemotional Changes Associated With HCWs Becoming Overweight or Obese From 2019 to 2022

Among the respondents, those who regularly exercised (539/8656, 6.23%) and consumed vegetables and fruit (546/8207, 6.65%) were less likely to have a change in BMI from underweight or normal weight to overweight or obese. Fewer respondents became overweight or obese from underweight or normal weight in those with a reduction or great reduction in their consumption of take-out food (369/3954, 9.33%), fried food (178/1623, 10.97%), snacks or desserts (221/2081, 10.62%), and sugar-sweetened beverages (192/1866, 10.29%); appetite (428/3427, 12.49%); alcohol consumption (84/882, 9.52%); persistent stress or recurrent anxiety or depression (819/10,584, 7.74%); and staying up late (740/9115, 8.12%) than the opposite. All of these were statistically significant (Table 4).

**Table 4:** Univariate analyses of lifestyle behavior and psychoemotional changes associated with health care workers becoming overweight or obese from 2019 to 2022.

Lifestyle behavior	Great increase/increase, n (%)	Great reduction/reduction, n (%)	*χ*² (*df*)	*P* value
Physical activity	539 (6.23)	470 (8.67)	33.367 (3)	<.001
Staying up late	740 (8.12)	245 (5.77)	28.326 (3)	<.001
Take-out food consumption	369 (9.33)	425 (7.59)	45.434 (3)	<.001
Vegetable and fruit consumption	546 (6.65)	291 (8.51)	14.875 (3)	.002
Breakfast frequency	306 (7.25)	263 (8.34)	8.170 (3)	.04
Fried food consumption	178 (10.97)	489 (6.67)	42.139 (3)	<.001
Snack or dessert consumption	221 (10.62)	435 (6.36)	48.245 (3)	<.001
Sugar-sweetened beverage consumption	192 (10.29)	406 (6.48)	43.987 (3)	<.001
Smoking	95 (9.24)	86 (6.66)	6.160 (3)	.10
Alcohol consumption	84 (9.52)	215 (6.9)	8.208 (3)	.04
Persistent stress or recurrent anxiety or depressed mood	819 (7.74)	138 (6.27)	9.657 (3)	.02
Sleep duration	200 (7.79)	721 (7.66)	9.126 (3)	.01
Appetite	428 (12.49)	197 (5.42)	173.898 (3)	<.001

### Multinomial Binary Logistic Regression Analyses of Factors Associated With HCWs Becoming Overweight or Obese From 2019 to 2022

Logistic regression analyses showed that the following were significant factors for BMI changes from underweight or normal weight in 2019 to overweight or obese in 2022: being 34-43 years (OR 0.843, 95% CI 0.740-0.960), 44-53 years (OR 0.738, 95% CI 0.635-0.960), and 54-63 years (OR 0.503, 95% CI 0.368-0.685) old; reduced physical activity (OR 1.201, 95% CI 1.055-1.368); and increased appetite (OR 2.043, 95% CI 1.788-2.034; Table 5).

**Table 5:** Multinomial binary logistic regression analyses of factors associated with health care workers becoming overweight or obese from 2019 to 2022.

Variables		OR (95% CI)	*P* value
Gender (reference: male)		1.095 (0.962-1.247)	.17
**Age (years; reference: 24-33 years)**			
	34-43	0.843 (0.740-0.960)	.01
	44-53	0.738 (0.635-0.857)	<.001
	54-63	0.503 (0.368-0.685)	<.001
	≥64	0.599 (0.301-1.193)	.15
**Education level** **(reference: junior high school or less)**			
	High school or technical secondary education	1.341 (0.677-2.658)	.40
	College or undergraduate degree	1.415 (0.717-2.794)	.32
	Master’s degree or higher	1.512 (0.724-3.159)	.27
**Main working and living location** **(reference: urban)**			
	Rural	1.015 (0.900-1.145)	.81
**Physical activity (reference: great increase/increase)**			
	Great reduction/reduction/no change/never or rarely	1.201 (1.055-1.368)	.006
**Staying up late (reference: great reduction/reduction/no change/never or rarely)**			
	Great increase/increase	1.017 (0.896-1.155)	.79
**Take-out food consumption (reference: great reduction/reduction/no change/never or rarely)**			
	Great increase/increase	1.056 (0.909-1.227)	.48
**Vegetable and fruit consumption (reference: great reduction/reduction/no change/never or rarely)**			
	Great increase/increase	0.968 (0.846-1.108)	.64
**Breakfast frequency (reference: great reduction/reduction/no change/never or rarely)**			
	Great increase/increase	0.938 (0.805-1.093)	.41
**Fried food consumption (reference: great reduction/reduction/no change/never or rarely)**			
	Great increase/increase	1.097 (0.875-1.375)	.42
**Snack or dessert consumption (reference: great reduction/reduction/no change/never or rarely)**			
	Great increase/increase	1.152 (0.928-1.431)	.20
**Sugar-sweetened beverage consumption (reference: great reduction/reduction/no change/never or rarely)**			
	Great increase/increase	0.918 (0.732-1.150)	.46
**Smoking (reference: great reduction/reduction/no change/never or rarely)**			
	Great increase/increase	1.140 (0.867-1.498)	.35
**Alcohol consumption (reference: great reduction/reduction/no change/never or rarely)**			
	Great increase/increase	0.999 (0.750-1.330)	.99
**Persistent stress or recurrent anxiety or depressed mood (reference: great reduction/reduction/no change/never or rarely)**			
	Great increase/increase	0.956 (0.847-1.080)	.47
**Appetite (reference: great reduction/reduction/no change)**			
	Great increase/increase	2.043 (1.788-2.334)	<.001
**Sleep duration (reference: great reduction/reduction/no change)**			
	Great increase/increase	0.961 (0.807-1.143)	.65

### Associations Between Persistent Stress or Recurrent Anxiety or Depressed Mood and Physical Exercise and Appetite

The results of the logistic regression analysis are shown in Table 6. Note that the sociodemographic characteristics were included as independent variables.

**Table 6:** Associations between persistent stress and/or recurrent anxiety/depressed mood (reference: reduction/no change/never or rarely) and physical activity and appetite.

Variables	Odds ratio (95% CI)	*P* value
Increased physical activity^a^	0.421 (0.398-0.447)	<.001
Increased appetite^b^	1.601 (1.483-1.728)	<.001

^a^Physical activity was the dependent variable.

^b^Appetite was the dependent variable.

## Discussion

This study investigated the self-rated health status, overweight and obesity rates, and lifestyle behavior changes of HCWs from 2019 to 2022 across China and analyzed the factors associated with weight changes from underweight or normal weight in 2019 to overweight or obese in 2022.

### Principal Findings

This study found that more HCWs reported a deteriorated health status than an improved status from 2019 to 2022. More than one-fifth reported having chronic diseases, which is significantly lower than that of a cross-China survey (34.29%) [55], and more of the chronic diseases improved rather than worsened, which is consistent with earlier findings in China [56].

The results demonstrated that, in 2019 and 2022, the overweight and obesity rates of HCWs were 43.1% and 45.48%, respectively, which is close to the survey in some Chinese cities among HCWs (44.98%) [57] but lower than that of the general population across China during the same period (49.6%) [58]. From 2019 to 2022, although the overweight and obesity rates among HCWs only increased by 2.38%, with an average annual growth rate that was slightly lower than that across China during the same period [58], the proportion of those changing from underweight or normal weight to overweight or obese was much higher than that of those changing from overweight or obese to underweight or normal weight.

This study showed that, from 2019 to 2022, the lifestyle behaviors of Chinese HCWs generally improved, and more had increased physical activity, vegetable and fruit consumption, and breakfast frequency as well as reduced consumption of take-out food, fried food, snacks or desserts, and sugary beverages as well as cigarette smoking.

Wang et al [59] conducted a study among doctors and nurses in north China in the same period and found that the top 5 predictors of a BMI change were an unbalanced diet, poor sleep quality, work-family conflict, lack of exercise, and soft drink consumption. This study’s univariate analyses revealed that increased physical activity and vegetable and fruit consumption could reduce the likelihood of going from underweight or normal weight to overweight or obese, while increasing the frequency of staying up late and consumption of take-out food, fried food, snacks or desserts, sugary beverages, and alcohol as well as appetite could increase the likelihood. These findings showed that lifestyle behavior changes are closely related to weight changes [60]. Multinomial binary logistic regression analyses demonstrated that increased appetite and reduced physical activity were significant risk factors for HCWs becoming overweight or obese from underweight or normal weight, suggesting that healthy dietary patterns and promotion of physical activity should be highlighted for the prevention of overweight or obesity among HCWs [61]. This study found that younger HCWs (24-33 years old) were more likely to become overweight or obese from underweight or normal weight than other age groups, which might be related to their increased involvement in frontline work in the pandemic [62].

An excessive workload can have a significant impact on lifestyle behaviors, resulting in becoming overweight or obese [63]. Studies have shown that the level of physical activity by HCWs was greatly reduced due to participating in pandemic prevention and control [64]. Liu et al [65] found that the risk of shift workers being overweight or obese increased by 25% and 17%, respectively. A study in China found that 48.5% of nurses developed sleep disorders during the COVID-19 pandemic due to overwork [66]. Mu et al [67] found that, during the pandemic, doctors slept significantly less. This survey found that, since the outbreak of COVID-19 in 2019, nearly 44.38% of HCWs stayed up late more or much more often and nearly 45.83% had a reduction or great reduction in sleep duration. Additionally, the relationship between sleep disorders and being overweight or obese has been confirmed by a substantial body of studies [68]. In this study, although univariate analysis found that an increased frequency of staying up late was statistically significantly associated with becoming overweight or obese, the increase was not a significant predictor in the logistic regression analysis. This discrepancy may stem from the fact that we only collected and analyzed changes in sleep duration (great increase/increase) without investigating and analyzing the actual sleep duration.

Increased stress and fear due to intensive work and a high risk of infection can have significant psychological and emotional effects [69-71]. A recent retrospective study revealed that, due to the impact of the COVID-19 pandemic, 40% of medical staff suffered from anxiety disorders and 37% suffered from depression disorders [72]. This survey discovered that persistent stress or recurrent anxiety or depressed mood among Chinese HCWs increased significantly, higher than the Chinese national average [73]. Psychological problems such as anxiety, depression, and stress can affect physical activity [74] and diet-related behaviors [75-77] and thus affect weight gain. Depression and obesity are reciprocal risk factors [78], and stress can lead to sleep disturbances [79], resulting in reduced physical activity and metabolic disorders [80]. This study demonstrated that increased persistent stress or recurrent anxiety or depressed mood were significantly associated with reduced physical activity and increased appetite. Therefore, providing psychological support for HCWs during the pandemic could not only benefit their mental health but could also reduce the possibility of becoming overweight or obese.

As far as the results of this study are concerned, an integrated approach is needed to address the problem of becoming overweight or obese and lifestyle changes among HCWs. At the national level, the right to rest and physical and mental health promotion should be ensured under a legal framework [81,82], especially in the event of a pandemic. At the institutional level, a system of rotational rest for HCWs should be established while ensuring institutional continuity. Second, regular psychological counselling and support services should be provided to ensure that stress and emotions are addressed in a timely manner [83,84]. Third, routine healthy lifestyle guidance (eg, by health coaches) and the modification of unhealthy dietary patterns and physical inactivity should be provided systematically and specifically [85]. Fourth, for those who are overweight or obese, lifestyle medicine intervention services should be given based on the principle of informed voluntary participation [86].

Notably, the impact of the COVID-19 epidemic on HCWs’ weight and lifestyle behaviors might be temporary. For instance, Oliver et al [11] found that, compared with before March 2020 (before the COVID-19 outbreak), in January 2021, nearly 70% of American HCWs experienced an increase in snacking, 60% consumed more fast food or take-out food, and 50% consumed more alcohol; however, by January 2022, the dietary habits of American HCWs returned to prepandemic patterns. More research is needed on the persistence of the impact of the pandemic on overweight and obesity rates and lifestyle behaviors of HCWs.

### Comparison With Prior Work

This study used a retrospective methodology to explore the weight gain status of Chinese HCWs during the pandemic period from December 2019 to August 2022, along with their psychoemotional and lifestyle exposures. To our knowledge, this is the first nationwide, large-scale study in China focusing on weight gain and influencing factors of HCWs during the pandemic. Compared with previous studies, its advantages are mainly manifested in 4 ways. First, this study adopted a 3-year retrospective cohort study design, making the conclusion on the correlation between the influencing factors and weight changes more reliable than previous studies using cross-sectional methods [13,32-34,66]. Second, although previous studies only analyzed the association between lifestyle behaviors and weight gain for HCWs during the pandemic [6,11], this study took lifestyle behaviors as both the independent variables of weight gain and the dependent variables of persistent stress or recurrent anxiety or depressed mood to conduct a correlational study, uncovering the significant effects of psychoemotional changes on lifestyle behaviors. Third, this study highlights the fundamental contributions of persistent stress or recurrent anxiety or depressed mood caused by high-risk and high-intensity work to weight gain rather than lifestyle behaviors [6,11,13], which were the mediating factors. Fourth, this study suggests that intervention and policy should focus on effectively alleviating intensive work-related stress to reduce the risk of weight gain for HCWs instead of only on lifestyle behavior interventions [6,13] during the pandemic.

### Limitations

This study had the following limitations. First, the data in this study were collected through online self-reporting; their accuracy largely depended on the respondents’ ability to understand and willingness to answer questions. They may have underestimated or exaggerated the truth. However, because the respondents are medical and health personnel with professional backgrounds, the information collected is accurate to a certain extent. Second, this study did not divide the respondents into frontline personnel and nonfrontline personnel for a comparative analysis; hence, the conclusions should be cautiously accepted. Third, recall bias might not be excluded due to the retrospective nature of the study. However, the sample size of this study is large enough to compensate for these limitations to a certain extent, and the conclusions of this study still have validity.

### Future Directions

This study provides a new perspective for understanding the impact of psychological stress on HCW weight gain during the pandemic period. Further research might be needed to distinguish the pattern of the effect of psychological stress on lifestyle behaviors and weight gain among the different nature of pandemic-related work (ie, frontline and nonfrontline) and occupational categories (such as doctors, nurses, and disease prevention and control personnel). Additionally, a nested case control study [87] design can be adopted to more effectively distinguish the intensity of the impact of psychological stress on different lifestyle behaviors and weight gain. Finally, it could be of great significance to conduct research on the effectiveness of different psychological support strategies for improving the lifestyles of HCWs during an epidemic.

### Conclusions

In summary, the pandemic had an impact on Chinese HCWs becoming overweight or obese due to lifestyle behavior changes, especially reduced physical activity and increased appetite related to increased persistent stress or recurrent anxiety or depressed mood caused by an excessive workload. An integrated approach is needed to address overweight and obesity rates and lifestyle changes among HCWs, by releasing negative psychoemotional states through workload reduction in future stressful life events.

## Data Availability

The data sets used or analyzed in this study are available from the corresponding author on reasonable request.

## Abbreviations

CHERRIES: Checklist for Reporting Results of Internet E-Surveys
HCW: health care worker
